# Breast cancer detection and classification via a robust deep learning approach

**DOI:** 10.1038/s41598-026-62255-2

**Published:** 2026-07-17

**Authors:** Magy Makram, Alber S. Aziz, Mary Monir Saeid, Mostafa Thabet Mohamed

**Affiliations:** 1 Computer Science Department, Faculty of Computers and Artificial Intelligence, Fayoum University, Fayoum, Egypt; 2 Computer Science Department, Faculty of Information Systems and Computer Science, October 6 University, Giza, Egypt; 3 Information Systems Department, Faculty of Computers and Artificial Intelligence, Fayoum University, Fayoum, Egypt; 4 Artificial Intelligence Department, Faculty of Information Systems and Computer Science, October 6 University, Giza, Egypt

**Keywords:** Breast cancer classification, Mammography, CBIS-DDSM, ResNet50, Inter-view attention fusion, Transfer learning, Grad-CAM, Cancer, Computational biology and bioinformatics, Health care, Mathematics and computing, Medical research

## Abstract

This work presents a leakage-controlled deep-learning framework for breast cancer classification using the CBIS-DDSM mammography archive. The proposed pipeline combines patient-level data partitioning before augmentation, a two-stage transfer-learning strategy based on ResNet50, and an Inter-View Attention Fusion (IVAF) module for adaptive fusion of paired craniocaudal (CC) and mediolateral oblique (MLO) feature maps. IVAF was modeled as a light-weighted convolutional gating strategy added after the last ResNet50 convolutional layer in order to create a weighted spatial-channel representation from the paired mammography images. In terms of the performance of the model under CBIS-DDSM held-out testing protocol, the entire model scored an accuracy of 97.12%, sensitivity of 96.44%, specificity of 97.68%, and AUC-ROC of 0.9876 based on the test results obtained on 6,117 images of 222 different patients. The average accuracy obtained using 100 random seeds was found to be 97.11% ± 0.18%.

## Introduction

 Cancer of the breast continues to be the leading cause of diagnosed cancer in women. In 2022, about 2.3 million new cases of female breast cancer were reported accounting for almost 1/4th of all female cancers^1^. Prognostic outcomes are heavily influenced by the stage of the disease where five-year survival is extremely good in localized stages but much worse in cases of metastatic breast cancer^2^. Mammography screening thus forms an integral part of detection strategies for the disease. However, the process of interpretation of mammography requires substantial time and expertise and can be hindered due to radiologist shortages, especially in developing nations^3^.

Convolutional neural networks have been demonstrated to perform well in mammography-related tasks when trained and tested in controlled settings through learning image representations from raw pixel intensities^4,5^. Among available mammography datasets, CBIS-DDSM is widely used as it contains digitized film-screen mammograms and annotations for lesions and pathologic labels.

Although CBIS-DDSM is frequently used in studies on deep learning, reproducibility largely relies on how dataset splitting, augmentation sequence, pre-processing, and evaluation procedures are described^6,7^. Datasets of mammograms might contain several mammographic images of the same patient, including various view angles, sides of the breast and lesion annotation. Thus, patient-level splitting prior to augmentation is a key methodological approach that allows minimizing the chance of using mammograms of the same patient in multiple folds, i.e., training, validation, and test datasets. This work applies patient-level splitting prior to data augmentation and describes splits to ensure reproducibility. The component-level ablation analysis and repeated-seeds analysis were applied to estimate the effect and robustness of the proposed framework under the considered CBIS-DDSM setting.

Clinical practice of mammographic screening implies joint interpretation of CC and MLO mammographic images since complementary projections could contribute to diagnostic information. Some researchers have explored multi-view mammography with deep learning; however, different fusion approaches are used in different model complexities, input data requirements and evaluation procedure. This work explores the compact CC-MLO attention-gated fusion approach and its transparent evaluation within the patient-level CBIS-DDSM.

Key contributions of this paper can be summarized as:

1. A partitioning scheme for patient-level mammograms prior to data augmentation such that images belonging to the same patient get assigned either to training set, validation set or test set, avoiding patient-level overlaps.
2. Two-stage ResNet50 transfer learning approach was developed and validated using component-wise ablation studies and seed repetition.
3. An Inter-View Attention Fusion (IVAF) module was proposed for adaptive fusion of paired CC and MLO feature maps using spatial and channel-wise gating instead of concatenation.
4. The approach was evaluated under a controlled protocol of CBIS-DDSM with test set performance results, ablation results, comparisons with reimplemented CNN baselines and qualitative Grad-CAM visualizations.

It is important to note that the novelty of the approach does not depend on ResNet50 model only. ResNet50 is used as an efficient feature extractor, while the key novelty of the approach is the lightweight IVAF module itself.

The rest of this article is structured as follows. In Sect.  "Related Work", we provide a literature review for the following aspects: CBIS-DDSM mammography classification; transfer learning methods; deep hybrid learning models; and multi-view mammography fusion techniques. Section "Methodology" covers our proposed method, which includes dataset division at patient level; ROI-based image preprocessing; data augmentation; ResNet50; IVAF module; and two-stage training process. Section "Results and Discussion" reports the experimental results, which include performance evaluation; comparison with baseline models; ablation study; multi-seed evaluation; confusion matrix; ROC analysis; and qualitative interpretation by Grad-CAM.

## Related work

Previous work on deep-learning mammography classification using CBIS-DDSM includes backbone evaluations^8^, transfer-learning pipelines^9,10^, hyperparameter-optimized architectures^11^, attention-augmented hybrid classifiers^12^, and hybrid diagnostic frameworks^13^. The following subsections summarize representative directions and position the proposed framework relative to them. Complementary cancer-classification studies have also explored machine-learning and neutrosophic models for breast cancer detection and clustering^14,15^, broader deep-learning-based cancer categorization^16^, feature-fusion approaches for skin lesion and cancer classification^17,18^, optimization-based lung-cancer classification^19^, and clustering-based skin cancer detection^20^. These studies support the relevance of machine-learning-based cancer analysis and feature fusion, but they do not directly address patient-level CC-MLO mammography fusion on CBIS-DDSM.

### Backbone evaluation

Das et al.^8^ compared several ImageNet-pretrained architectures on CBIS-DDSM under a shared augmentation and sampling setting. Their work helped establish the value of deeper ImageNet-pretrained CNN backbones for mammographic representation learning. It also illustrates why transparent reporting of splitting, augmentation order, and evaluation protocol is important when comparing CBIS-DDSM results across studies.

### Transfer-learning technique

Coto Santiesteban et al.^9^ applied dense layers at the end of VGG16 model and contrast-limited adaptive histogram equalization for preprocessing. Falconi et al.^10^ studied the effect of VGG16/VGG19 fine-tuning depth and showed that selective freezing provides better generalization than full unfreezing. Thus, there is an opportunity to implement a transfer learning process in stages for mammography classification if the target data set is smaller than the natural images that were used for CNN pre-training.

### Hybrid architectures

Houssein et al.^11^ proposed the combination of ResNet50 and the improved marine predators algorithm optimizer for hyperparameter tuning and class balancing. Chakravarthy et al.^12^ suggested ESA-XGBNet that combines EfficientNet-B0, spatial attention mechanism, XGBoost classifier, and Grad-CAM visualization. Murty et al.^13^ developed a hybrid deep learning approach for breast cancer classification based on CBIS-DDSM and Wisconsin Breast Cancer Database data sets. The aforementioned works cover the wide range of CNN-based and hybrid techniques for mammography classification, but numerical comparison of their results is possible only if data splits and preprocessing techniques are similar.

### Fusion methodology

Because mammograms are routinely acquired in CC and MLO views, multi-view fusion has become an important research direction. A common strategy is to process each view using shared or parallel CNN branches and then combine the resulting feature maps by concatenation or interaction-based fusion^8,21^. More recent graph-, transformer-, and hypercomplex-based approaches model relationships across views more explicitly^22–25^. The proposed IVAF module follows a lighter design by learning attention-gated CC-MLO fusion weights after ResNet50 feature extraction.

### Multi-view mammography classification

Recent mammography classification studies increasingly emphasize integration of multiple mammographic views rather than isolated single-image analysis. Early high-resolution multi-view CNNs showed that exam-level processing of multiple views can improve screening-oriented classification^22^. Transformer-based methods have also been proposed to model long-range interactions among unregistered mammographic views^23^, while hypercomplex learning has been used to capture correlations among views through parameterized multi-view representations^24^. Manigrasso et al. systematically investigated graph- and transformer-based multi-view architectures for mammography classification, highlighting the relevance of structured multi-view modeling^25^.

Compared with these approaches, IVAF is designed as a compact attention-gated fusion module for paired CC and MLO feature maps. Direct numerical comparison with graph-, transformer-, or hypercomplex-based methods should be interpreted cautiously because datasets, label definitions, preprocessing protocols, input resolutions, and evaluation settings may differ.

**Table 1:** Performance of traditional CNN backbones on CBIS-DDSM.

Model	Year	F1 score (%)	Citations
VGG19	2023	78.6	^8^
ResNet50	2023	83.9	^8^
MobileNetV2	2023	78.7	^8^
InceptionV3	2023	88.0	^8^
Xception	2023	89.5	^8^

**Table 2:** Comparison of advanced and transfer-learning models on CBIS-DDSM.

Model	Year	Accuracy (%)	F1 score (%)	AUC	Citations
VGG16-C300	2025	–	–	0.80	^9^
VGG16-8-FT	2020	84.4	85.0	0.844	^10^
IMPA-ResNet50	2022	98.32	97.65	0.979	^11^
ESA-XGBNet	2024	97.59	–	–	^12^
ILB-BCD	2024	99.16	–	–	^13^

Recent mammography studies have also investigated ResNet50 with balancing strategies^26^, EfficientNet-based augmentation and tailored architectures^27^, lightweight EfficientNet-B3 variants^28^, comparative deep-learning baselines^29^, and multi-model feature ensembles^30^. Together, these studies further show that breast cancer classification performance depends strongly on the selected backbone, feature representation, balancing strategy, and evaluation design.

**Table 3:** Representative multi-view mammography classification studies and relation to IVAF.

Study	Main approach	Multi-view strategy	Relation to IVAF
Geras et al.^23^	High-resolution multi-view CNN	Processes multiple mammographic views at exam level	Demonstrates exam-level multi-view screening; IVAF focuses on lightweight paired CC-MLO fusion.
Chen et al.^24^	Transformer-based mammography diagnosis	Combines unregistered mammographic views using transformer modeling	Uses global transformer interactions; IVAF uses compact convolutional attention gating.
Lopez et al.^25^	Hypercomplex multi-view learning	Models correlations among views using hypercomplex representations	Captures view relations through hypercomplex learning; IVAF learns spatial/channel-wise CC-MLO weights.
Manigrasso et al.^26^	Graph- and transformer-based multi-view learning	Investigates structured relationships among mammographic views	Models graph/transformer view relationships; IVAF is a compact ResNet50-based cross-view module.
Proposed ResNet50 + IVAF	Two-stage ResNet50 with IVAF	Learns adaptive attention-gated fusion between CC and MLO feature maps	Designed for a leakage-controlled CBIS-DDSM protocol with ablation and repeated-seed evaluation.

Tables 1, 2 and 3 contextualize the proposed framework relative to representative CNN, transfer-learning, hybrid, and multi-view mammography studies. Direct numerical comparison should be interpreted cautiously because published studies may differ in dataset partitions, preprocessing pipelines, label definitions, image resolution, and evaluation protocols.

## Methodology

**Fig. 1:**
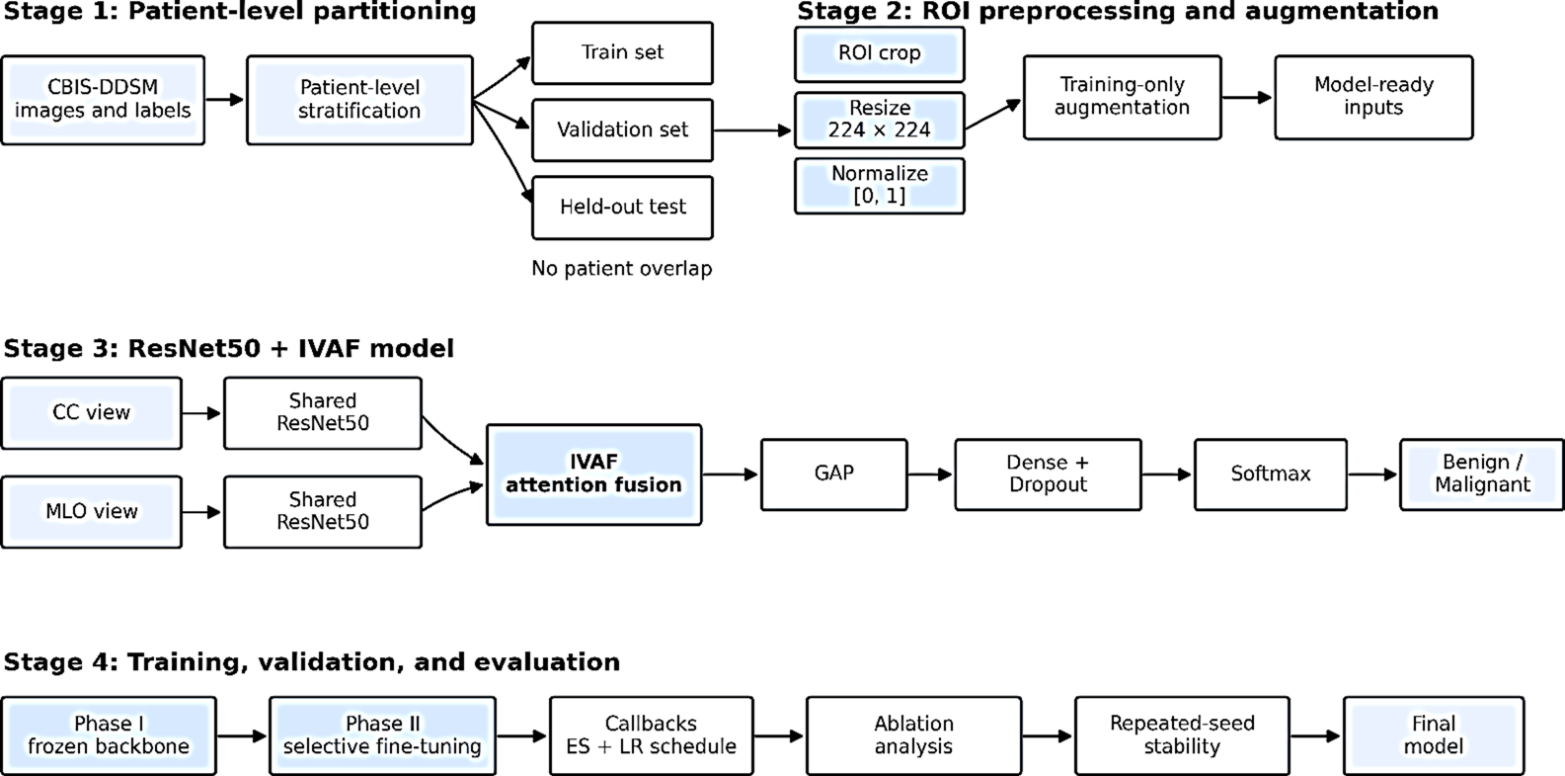
End-to-end workflow of the proposed ResNet50 + IVAF pipeline.

The proposed diagnostic pipeline consists of four main blocks: (1) patient-level data partitioning before augmentation; (2) ROI-guided preprocessing and training-only augmentation; (3) a ResNet50 backbone coupled with the proposed IVAF module; and (4) a two-stage transfer-learning procedure with regularization callbacks. The implementation used TensorFlow 2.12/Keras 2.12 and was run on a single NVIDIA A100 GPU with 80 GB VRAM. The overall training time took approximately 4.2 h with 1.8 h for Phase I and 2.4 h for Phase II, considering that a mini-batch size of 32 and the Adam optimizer, which consists of $\:{\beta\:}_{1}=0.9$, $\:{\beta\:}_{2}=0.999$, and $\:\epsilon = 1 \times \:10^{{ - 7}}$., were employed. The overall procedure can be seen in Fig. 1.

The ResNet50 network has been chosen as the backbone for extracting features due to two reasons: first, residual connections make the optimization process more stable in deep CNN models; second, this architecture is a standard one when it comes to medical image classification using transfer learning. In this way, we obtain a unified feature extractor for CC and MLO pairs without paying too much attention to the backbone.

### Patient-level data stratification and leakage control

Isolation of patient-level data prior to preprocessing and augmentation is the critical methodology in this study. Patient-level partitioning is significant in mammographic imaging datasets due to the fact that several images, views, lateralities, and annotations can belong to one and the same patient. The inclusion of all images of one patient in one and the same partition ensures that patient-specific anatomical information does not find its way into training and test splits simultaneously.

In this study, 1,109 distinct patients were stratified based on pathological class and assigned to non-overlapping training, validation, and test partitions prior to any transformations of images. The training set consisted of 665 patients, the validation set consisted of 222 patients, while the test set consisted of 222 patients. Augmentation was applied only to images in the training set, and hence the training dataset increased from 18,120 samples to 50,025 samples. Validation and test sets remained unaltered throughout the process. Partition statistics are provided in Table 4.

**Table 4:** Patient-level CBIS-DDSM partition statistics.

Partition	Unique patients	Original images	Augmented images	Benign (original)	Malignant (original)
Training	665	18,120	50,025	9,965 (55%)	8,155 (45%)
Validation	222	6,048	6,048	3,326 (55%)	2,722 (45%)
Test	222	6,117	6,117	3,366 (55%)	2,751 (45%)
Total	1,109	30,285	62,190	16,657	13,628

### Image preprocessing

The mammography images were resampled to 224 × 224 pixels via bicubic interpolation for the sake of meeting the spatial input constraint of the ResNet50 backbone network architecture, while preserving the texture information at the level of lesions. The focal lesion was isolated from the high resolution mammography image via the use of ROI masks from CBIS-DDSM and then resampling was done. These preprocessing steps guide the input of the model towards annotated lesion areas while maintaining the surroundings context.

The pixel intensities were scaled to the continuous range $\:\left[0,1\right]$ according to:

1 $$\:{I}_{\mathrm{n}\mathrm{o}\mathrm{r}\mathrm{m}}\left(x,y\right)=\frac{{I}_{\mathrm{o}\mathrm{r}\mathrm{i}\mathrm{g}\mathrm{i}\mathrm{n}\mathrm{a}\mathrm{l}}\left(x,y\right)}{255}.\:\:$$

Here, $\:{I}_{\mathrm{o}\mathrm{r}\mathrm{i}\mathrm{g}\mathrm{i}\mathrm{n}\mathrm{a}\mathrm{l}}\left(x,y\right)$ represents the original pixel intensity at coordinates $\:\left(x,y\right)$. The process of linear scaling ensures that the input intensity values are standardized for consistent optimization while maintaining the relative contrast relationship within the mammogram image.

**Fig. 2:**
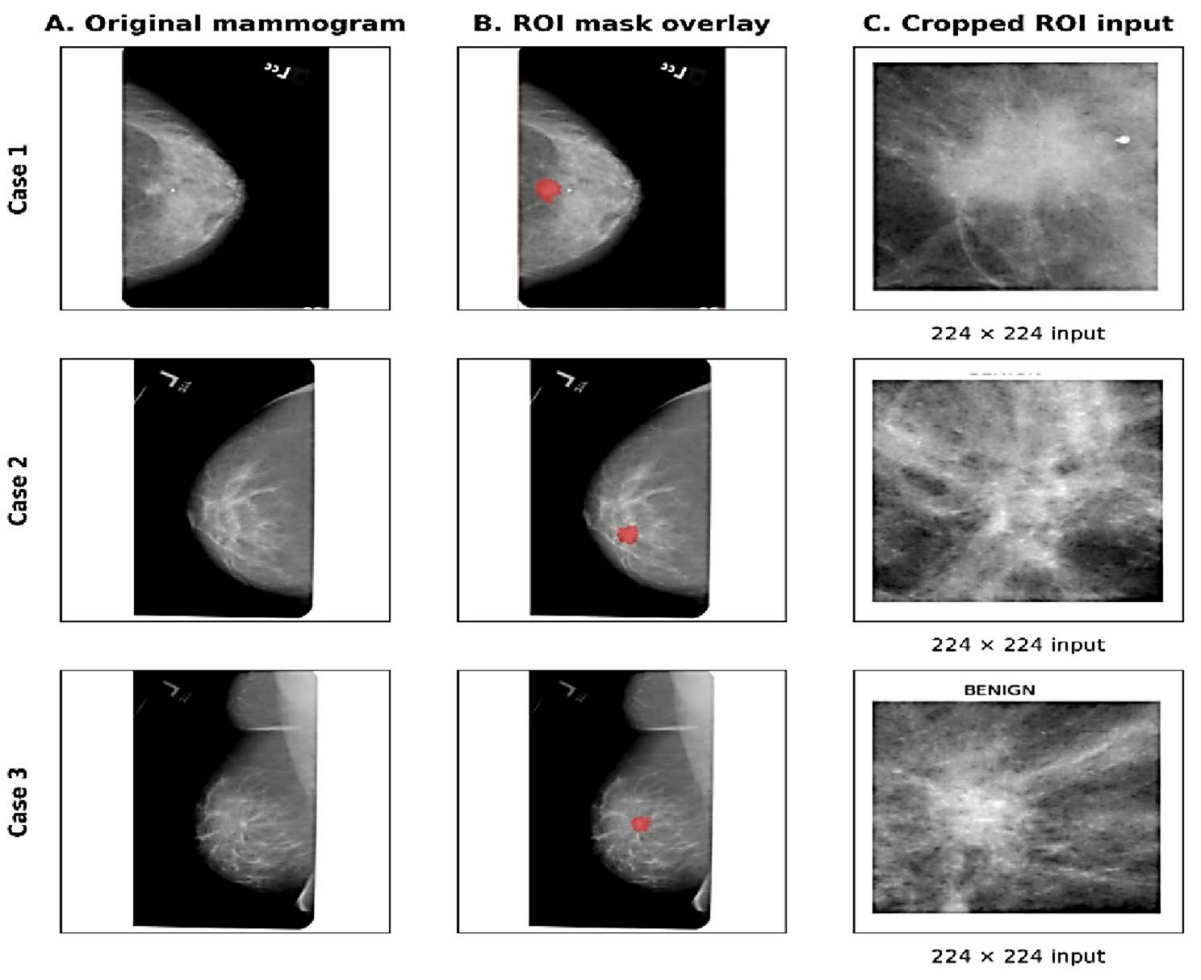
ROI-guided preprocessing workflow.

### Data augmentation

In order to introduce variability in the training set and resolve class imbalance, stochastic geometric and photometric transformations were performed only on the training data following patient-level stratification. This step ensured that augmented training images did not contain patient-specific anatomical information common to validation or testing images. These augmentations comprised in-plane rotations between$\:-{15}^{\circ\:}$and $\:+{15}^{\circ\:}$, random horizontal and vertical translations up to $\:\pm\:10\mathrm{\%}$, isotropic scaling between $\:0.90$ to $\:1.10$, anatomically realistic flipping, and contrast/brightness adjustments of $\:\pm\:20\mathrm{\%}$^31^.

All these transformations were restricted to maintain the lesion structure and avoid clinically implausible artifacts. The overall transformation procedure for each training image can be mathematically defined as:

2 $$\:{I}_{\mathrm{a}\mathrm{u}\mathrm{g}}={T}_{\mathrm{c}\mathrm{o}\mathrm{m}\mathrm{p}\mathrm{o}\mathrm{s}\mathrm{i}\mathrm{t}\mathrm{e}}\left({I}_{\mathrm{o}\mathrm{r}\mathrm{i}\mathrm{g}\mathrm{i}\mathrm{n}\mathrm{a}\mathrm{l}}\right).\:\:$$

whereas $\:{T}_{\mathrm{c}\mathrm{o}\mathrm{m}\mathrm{p}\mathrm{o}\mathrm{s}\mathrm{i}\mathrm{t}\mathrm{e}}$ stands for the stochastic composition of the active transformations. The post-split augmentation was used in conjunction with the class-weighted loss according to Sect "Class-imbalance mitigation via balanced loss weighting".

### Model architecture

#### ResNet50 backbone and classification head

Each mammographic view is processed by a ResNet50 backbone initialized with ImageNet weights. The residual connection in each block is summarized by:

3 $$\:y=F\left(x,\left\{{W}_{i}\right\}\right)+x.\:\:$$

where $\:x$ denotes the input to the block, $\:F\left(x,\left\{{W}_{i}\right\}\right)$ is is the residual mapping with parameters $\:\left\{{W}_{i}\right\}$, represented by convolutional filters, and $\:y$ is the output from the block.

Following feature extraction and IVAF fusion, Global Average Pooling turns the fused feature tensor into a compact channel-wise feature representation. Next, a Dropout layer with 0.5 dropout probability, followed by a dense layer of 1,024 neurons and ReLU activations and finally a Softmax layer are used for classifying benign/malignant. Standard CNN layers are concisely reported as our major contributions lie elsewhere in the methodology.

**Fig. 3:**
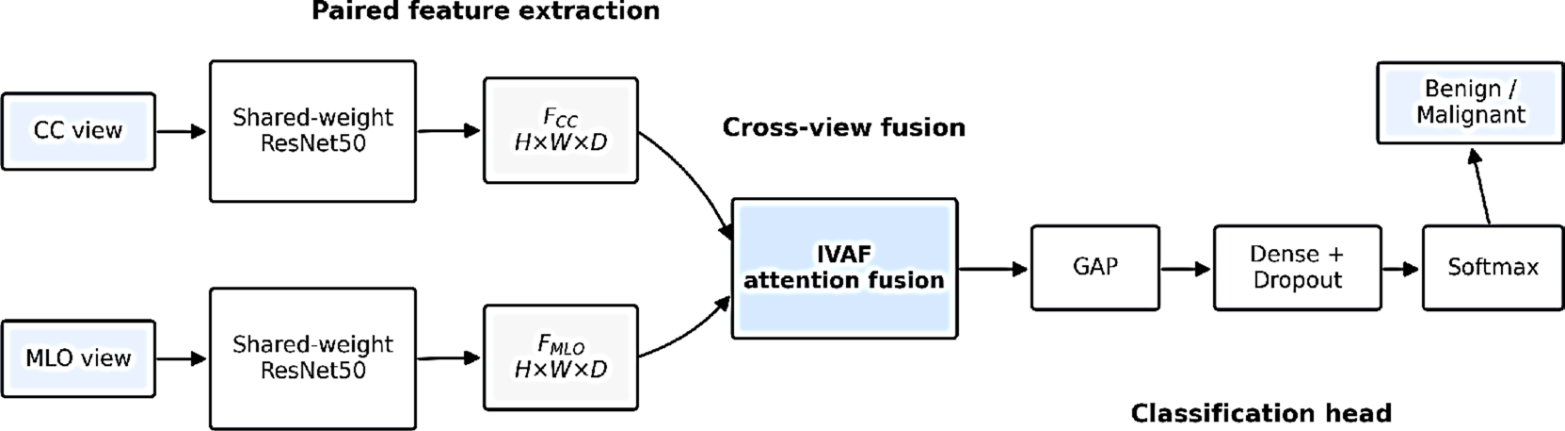
Overview of the proposed ResNet50 + IVAF architecture.

#### Inter-view attention fusion (IVAF) module

IVAF module is designed such that two types of complementary CC and MLO views are merged using adaptive attention mechanism, unlike giving equal weightage to both the views. The reason behind such a choice is that complementary information from both the views may add up to help in achieving better accuracy in classification.

**Fig. 4:**
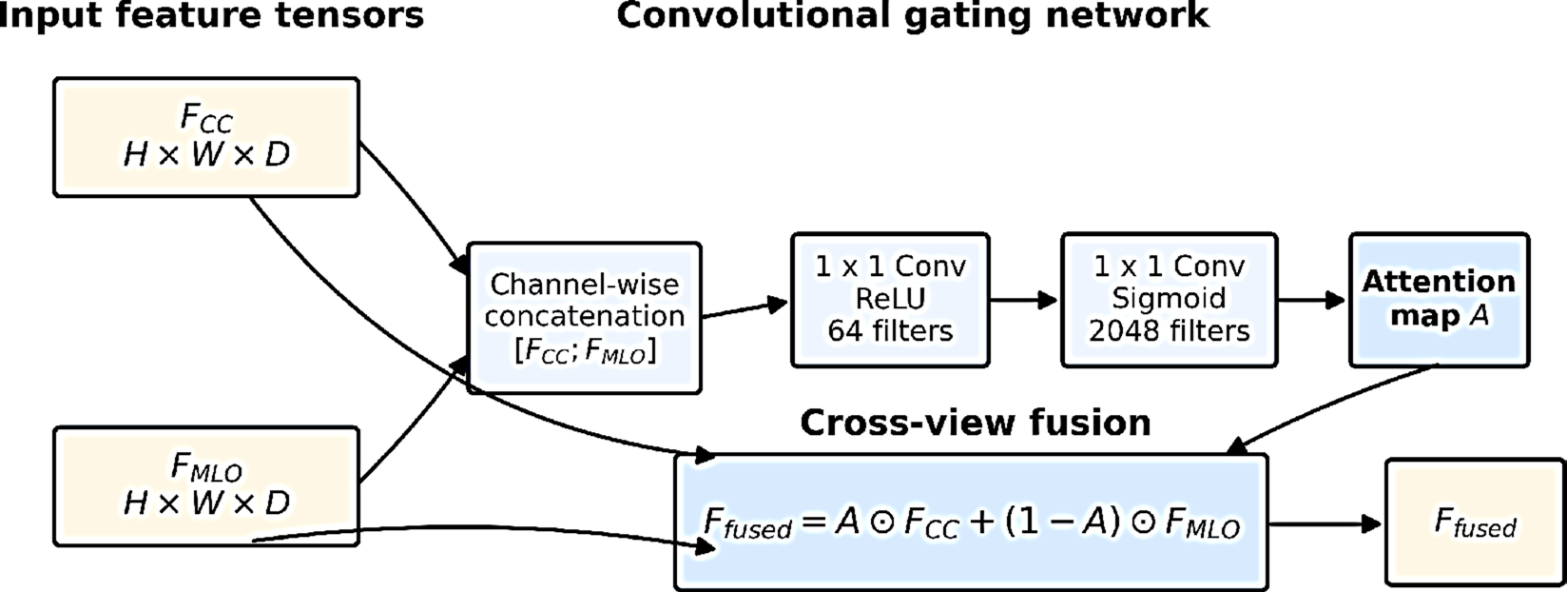
Enhanced IVAF module architecture.

In Fig. 4, it can be seen that IVAF takes feature tensors from paired mammographic views extracted using ResNet50 branches of shared weights. This helps in mapping both the views to the same feature space for feature comparison prior to merging:

4 $$\:{F}_{CC},{F}_{MLO}\in\:{\mathbb{R}}^{H\times\:W\times\:D}.\:\:$$

The feature tensors are concatenated along the channel dimension:

5 $$\:{F}_{\mathrm{c}\mathrm{a}\mathrm{t}}=\left[{F}_{CC};{F}_{MLO}\right].\:\:$$

The attention map is produced using a lightweight gated convolutional network based on a 1 × 1 convolutional layer with ReLU activation and 64 filters, and a second 1 × 1 convolutional layer with 2,048 filters and sigmoid activation:

6 $$\:A=\sigma\:\left({\mathrm{C}\mathrm{o}\mathrm{n}\mathrm{v}}_{1\times\:1}^{2048}\left(\mathrm{R}\mathrm{e}\mathrm{L}\mathrm{U}\left({\mathrm{C}\mathrm{o}\mathrm{n}\mathrm{v}}_{1\times\:1}^{64}\left({F}_{\mathrm{c}\mathrm{a}\mathrm{t}}\right)\right)\right)\right),\:A\in\:{\mathbb{R}}^{H\times\:W\times\:D}.\:\:$$

The final fused feature tensor is computed as:

7 $$\:{F}_{\mathrm{f}\mathrm{u}\mathrm{s}\mathrm{e}\mathrm{d}}=A\odot\:{F}_{CC}+\left(1-A\right)\odot\:{F}_{MLO}.\:\:$$

where ⊙ represents the Hadamard product of tensors. The combined tensor $\:{F}_{\mathrm{f}\mathrm{u}\mathrm{s}\mathrm{e}\mathrm{d}}$ is then passed on to the Global Average Pooling layer and the classification layer. The above formulation allows for learning of adaptive contributions of CC-MLO at each spatial position and channel. Classification can be formulated as:

8 $$\:\widehat{y}=\mathrm{S}\mathrm{o}\mathrm{f}\mathrm{t}\mathrm{m}\mathrm{a}\mathrm{x}\left({W}_{2}\hspace{0.17em}\mathrm{R}\mathrm{e}\mathrm{L}\mathrm{U}\left({W}_{1}\hspace{0.17em}\mathrm{G}\mathrm{A}\mathrm{P}\left({F}_{\mathrm{f}\mathrm{u}\mathrm{s}\mathrm{e}\mathrm{d}}\right)+{b}_{1}\right)+{b}_{2}\right).\:\:$$

where $\:{W}_{1}$, $\:{W}_{2}$, $\:{b}_{1}$, and $\:{b}_{2}$ denote trainable classification-head parameters and $\:\widehat{y}$ is the predicted class-probability vector.

### Two-stage transfer-learning strategy

A two-stage training regimen was implemented to address the domain shift from natural images to mammographic images, following common transfer-learning practice in medical image classification^32^. In Stage I, the ResNet50 backbone was frozen and only the randomly initialized classification head was trained. The Stage I update is expressed as:

9 $$\:{\theta\:}_{\mathrm{h}\mathrm{e}\mathrm{a}\mathrm{d}}^{\left(t+1\right)}={\theta\:}_{\mathrm{h}\mathrm{e}\mathrm{a}\mathrm{d}}^{\left(t\right)}-{\eta\:}_{1}{\nabla\:}_{{\theta\:}_{\mathrm{h}\mathrm{e}\mathrm{a}\mathrm{d}}}L\left({\theta\:}_{\mathrm{h}\mathrm{e}\mathrm{a}\mathrm{d}},{\theta\:}_{\mathrm{b}\mathrm{a}\mathrm{c}\mathrm{k}\mathrm{b}\mathrm{o}\mathrm{n}\mathrm{e}}^{\mathrm{f}\mathrm{r}\mathrm{o}\mathrm{z}\mathrm{e}\mathrm{n}}\right).\:\:$$

The initial learning rate was set to $\:{\eta\:}_{1}=1\times\:{10}^{-4}$. Freezing the backbone preserves pretrained low- and mid-level feature hierarchies while allowing the classification head to adapt to the mammography label space.

In Stage II, the final two residual blocks of ResNet50 were unfrozen and fine-tuned with the classification head using a lower learning rate of $\:{\eta\:}_{2}=1\times\:{10}^{-5}$:

10 $$\:{\theta\:}^{\left(t+1\right)}={\theta\:}^{\left(t\right)}-{\eta\:}_{2}{\nabla\:}_{\theta\:}L\left({\theta\:}_{\mathrm{h}\mathrm{e}\mathrm{a}\mathrm{d}},{\theta\:}_{\mathrm{b}\mathrm{a}\mathrm{c}\mathrm{k}\mathrm{b}\mathrm{o}\mathrm{n}\mathrm{e}}\right).\:\:$$

Selective fine-tuning was used because earlier convolutional layers capture generic low-level features, whereas deeper layers encode more task-specific representations^13,29^. The lower Stage II learning rate reduces the risk of destabilizing useful pretrained features.

### Class-imbalance mitigation via balanced loss weighting

CBIS-DDSM is moderately imbalanced, with benign lesions representing approximately 55% of the original dataset. To reduce bias toward the majority class, class-balanced categorical cross-entropy was used during both training stages:

11 $$\:L_{{{\mathrm{weighted}}}} = - \frac{1}{N}\sum\limits_{{i = 1}}^{N} {\sum\limits_{{C = 1}}^{C} {w_{c} y_{{i,c}} {\mathrm{log}}\left( {\hat{y}_{{i,c}} } \right)} }$$

The class weight for class $\:c$ was computed as:

12 $$\:{w}_{c}=\frac{N}{C\hspace{0.17em}{N}_{c}}.\:\:$$

where $\:N$ is the number of training samples, $\:C$ is the number of classes, and $\:{N}_{c}$ is the number of samples belonging to class $\:c$. This weighting strategy encourages each class to contribute more evenly to the total loss and is commonly used to reduce imbalance-related bias in medical image classification^33^.

### Training callbacks and regularization

Two adaptive callbacks were used during training. EarlyStopping monitored the validation loss and restored the best weights when improvement plateaued. The stopping condition can be written as:

13 $$\:{\mathrm{Stopif}}v_{t} \ge \:{\mathrm{min}}\left( {v_{{t - \delta \:}} , \ldots \:,v_{{t - 1}} } \right) - \epsilon ,\,\,\,\,\,\,\,\,\,\,\,\:\:\delta \: = 5,\,\,\epsilon = 10^{{ - 4}} .\:\:$$

where $\:{v}_{t}$ is the validation loss at epoch $\:t$. ReduceLROnPlateau reduced the learning rate when validation loss stopped improving:

14 $$\:{\eta\:}_{t+1}=\left\{\begin{array}{ll}0.5{\eta\:}_{t},&\:\mathrm{i}\mathrm{f}\hspace{0.25em}\mathrm{p}\mathrm{l}\mathrm{a}\mathrm{t}\mathrm{e}\mathrm{a}\mathrm{u};\\\:{\eta\:}_{t},&\:\mathrm{o}\mathrm{t}\mathrm{h}\mathrm{e}\mathrm{r}\mathrm{w}\mathrm{i}\mathrm{s}\mathrm{e}.\end{array}\right.\:\:$$

Together with Dropout, class-balanced loss weighting, EarlyStopping, and learning-rate reduction were used to regularize optimization and reduce overfitting.

## Results and discussion

### Test-set classification performance

The complete ResNet50 + IVAF network trained using the two-stage strategy on patient-level CBIS-DDSM partitions achieved an overall accuracy of 97.12% on the held-out test set of 6,117 images from 222 unseen patients. Evaluation was performed on an unaugmented test partition with no patient-level overlap with the training or validation sets. The model achieved sensitivity of 96.44%, specificity of 97.68%, and AUC-ROC of 0.9876. Full per-class precision, recall, F1-score, and support values are reported in Table 5.

**Table 5:** Per-class and aggregate performance on the CBIS-DDSM test set.

Class	Precision	Recall	F1-score	Support
Benign (0)	97.11%	97.68%	97.39%	3,366
Malignant (1)	97.14%	96.44%	96.79%	2,751
Overall accuracy	-	-	97.12%	6,117
Macro average	97.12%	97.06%	97.09%	6,117
Weighted average	97.12%	97.12%	97.12%	6,117

### Ablation study and component-wise contribution analysis

To evaluate the contribution of each component, an ablation study was performed in which components were added sequentially to the baseline configuration while keeping the data split, preprocessing, optimizer, batch size, learning rates, computing device, and test protocol fixed. The results are reported in Table 6.

The baseline configuration, consisting of a frozen ResNet50 backbone and a randomly initialized classification head, achieved an accuracy of 93.42%. Selective fine-tuning improved accuracy by 1.86% points. Adding class-balanced loss weighting improved both sensitivity and specificity, and applying post-split augmentation further improved performance. The IVAF module produced an additional improvement under the same experimental conditions. The IVAF-related improvement was statistically significant according to McNemar’s test ($\:{\chi\:}^{2}=8.91$, $\:p=0.003$), although it should be interpreted as a modest but meaningful improvement within the evaluated CBIS-DDSM setting rather than as evidence of broad clinical generalizability.

**Table 6:** Ablation study showing incremental contribution of pipeline components.

Model configuration	Accuracy	Delta accuracy	Sensitivity	Specificity
A. Baseline: Phase I frozen backbone only	93.42%	–	92.89%	93.78%
B. A + Phase II selective fine-tuning	95.28%	+ 1.86 pp	94.75%	95.68%
C. B + class-balanced loss weighting	96.17%	+ 0.89 pp	95.68%	96.51%
D. C + post-split data augmentation	96.73%	+ 0.56 pp	96.10%	97.18%
E. D + IVAF module (full model)	97.12%	+ 0.39 pp	96.44%	97.68%

### Training dynamics and convergence analysis

**Fig. 5:**
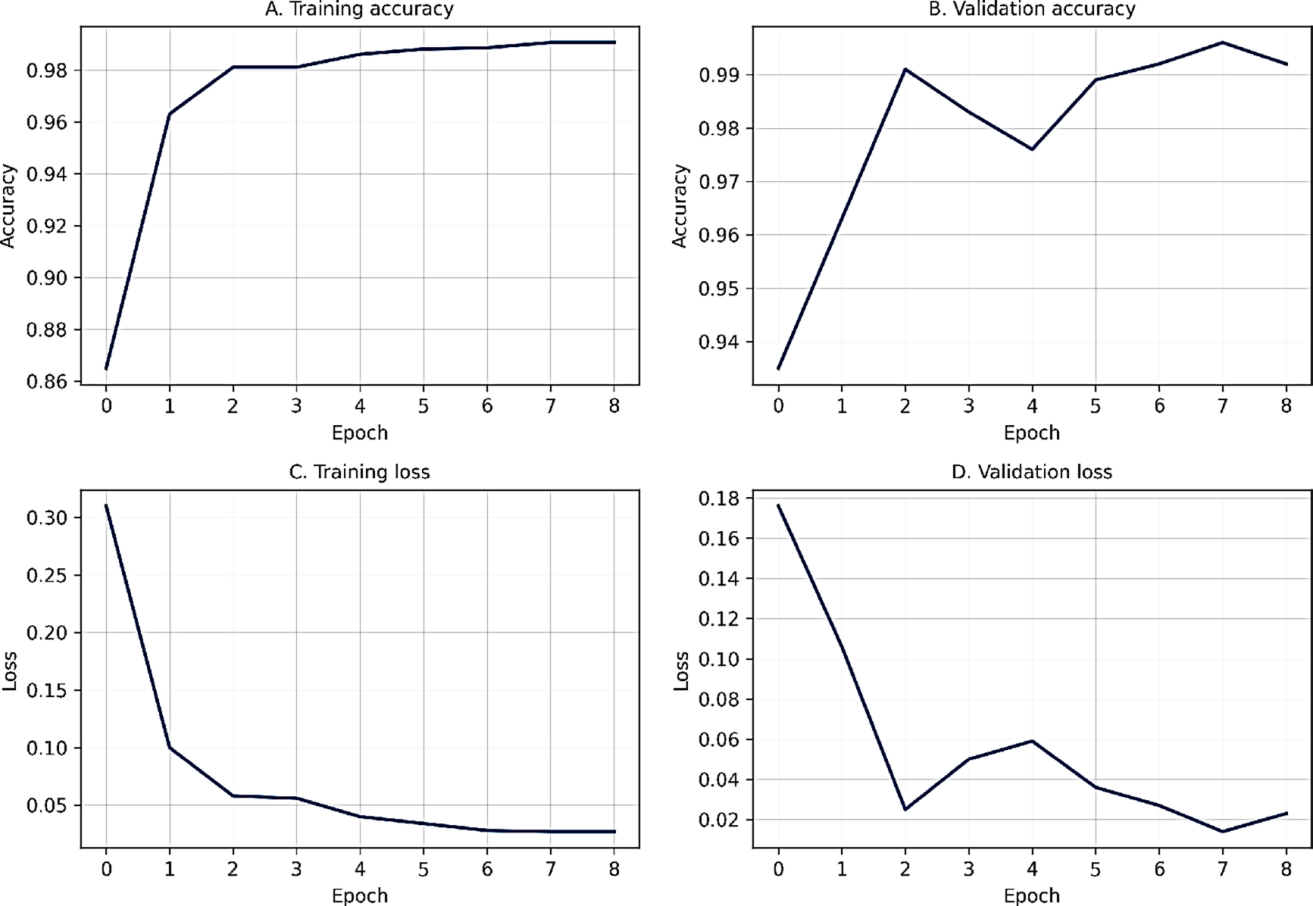
Training and validation learning curves.

Figure 5 presents training dynamics that demonstrate a stable convergence without any observable divergence between training and validation loss. To analyze sensitivity to the initial weights, we have performed training using 100 different random seeds on the same patient-level split of the data. The mean ± standard deviation across repeated runs was accuracy = 97.11% ± 0.18%, sensitivity = 96.43% ± 0.29%, specificity = 97.66% ± 0.27%, and AUC-ROC = 0.9874 ± 0.0012. These results indicate stable performance under the evaluated training protocol.

### Comparison with reimplemented CNN architectures

For the purpose of benchmarking the methodology used, seven architectures were replicated in a controlled experimental environment using the CBIS-DDSM dataset, including the use of patient-level splitting, similar preprocessing and augmentation steps, the same two-phase training regime, Adam optimizer, batch size of 32, NVIDIA A100 GPU, and the same unaugmented test set.

In this experiment, ResNet50 + IVAF performed better in terms of accuracy and AUC-ROC compared to the other replications, where its accuracy is 97.12%, sensitivity is 96.44%, specificity is 97.68%, and AUC-ROC is 0.9876. The result of this comparison should be taken as an internal benchmark within the controlled environment rather than being a claim of superiority against other published mammography architectures.

**Table 7:** Controlled comparison against reimplemented CNN architectures.

Architecture	Accuracy	Sensitivity	Specificity	AUC-ROC	Inference time (ms)
VGG16	95.82%	94.92%	96.31%	0.9712	42.1
VGG19	95.45%	93.78%	96.48%	0.9689	48.6
ResNet101	96.84%	96.11%	97.19%	0.9851	35.2
EfficientNet-B0	96.31%	95.67%	96.71%	0.9814	19.3
EfficientNet-B3	96.78%	95.95%	97.28%	0.9843	26.7
DenseNet121	96.56%	95.88%	97.01%	0.9828	31.8
MobileNetV2	93.47%	91.83%	94.76%	0.9534	14.2
ResNet50 + IVAF	97.12%	96.44%	97.68%	0.9876	28.4

### Confusion matrix and ROC curve analysis

**Fig. 6:**
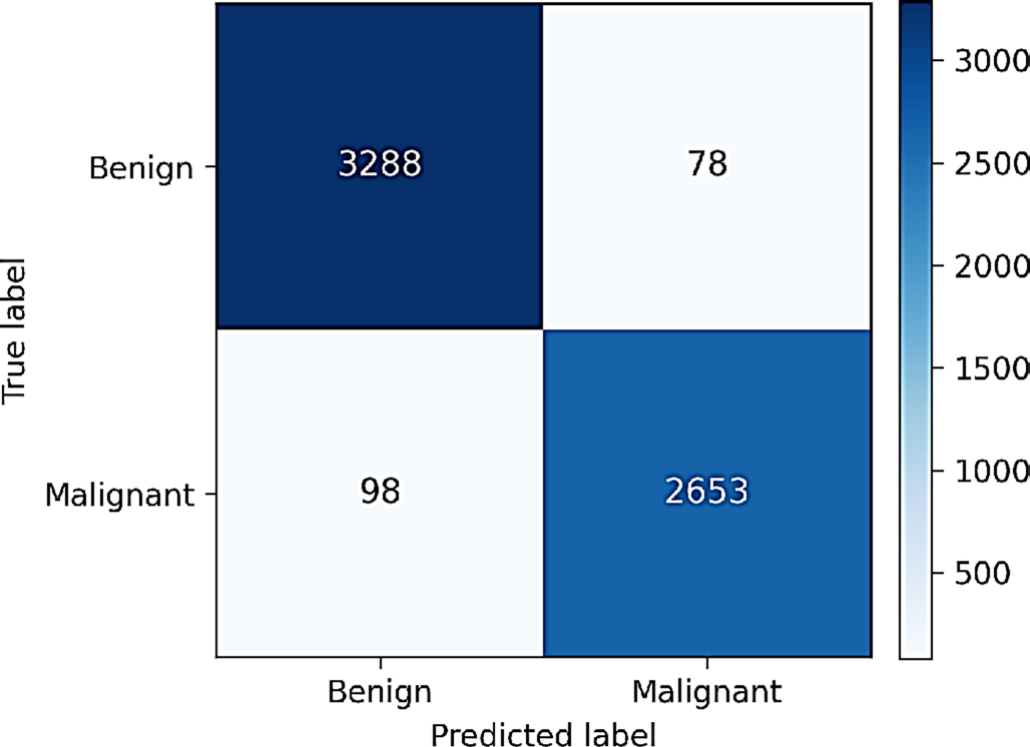
Confusion matrix on the held-out test set.

**Fig. 7:**
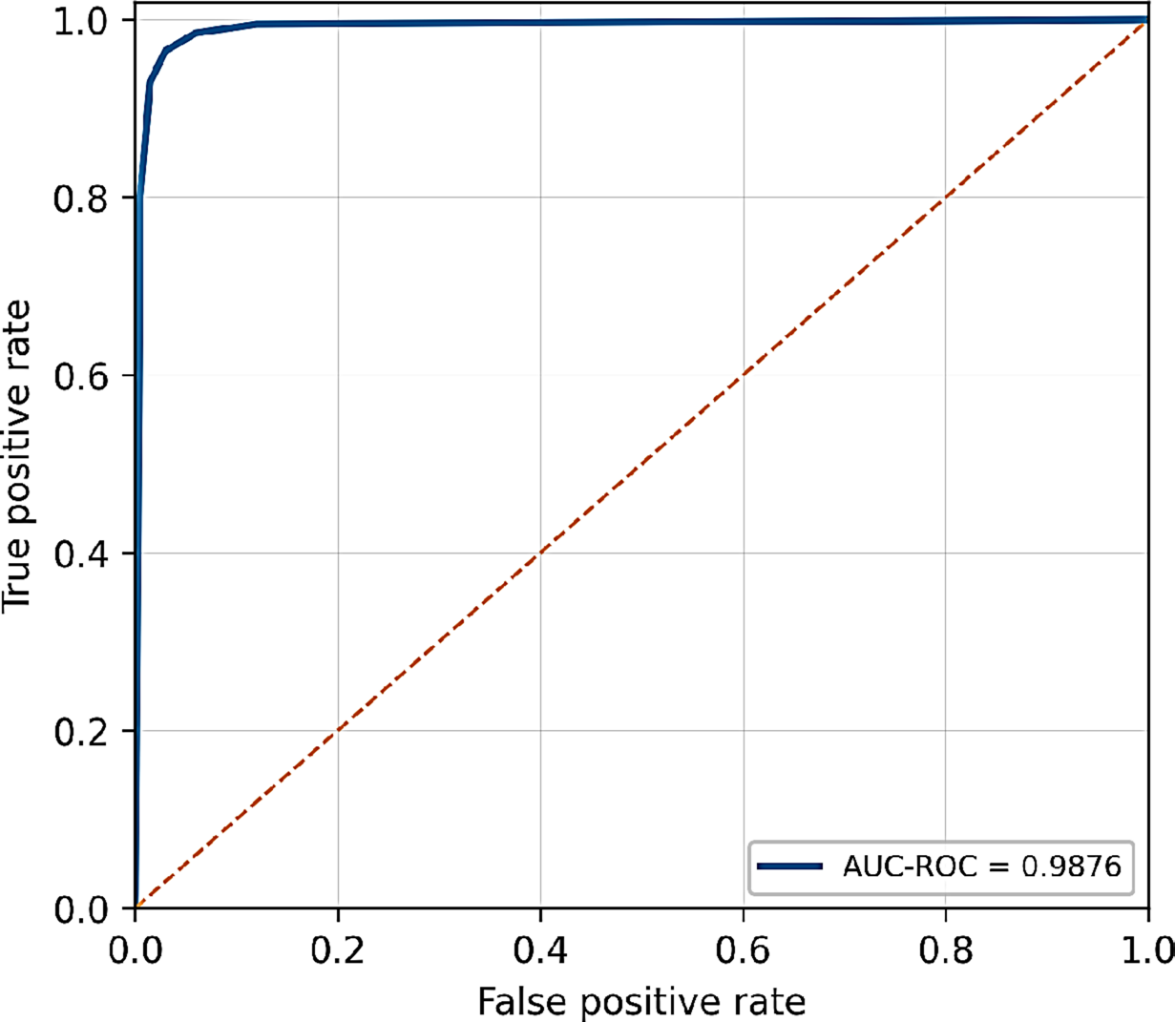
ROC curve on the held-out test set.

Figure 6 presents the confusion matrix for the held-out CBIS-DDSM test set. The matrix contains 3,288 correctly classified benign images, 2,653 correctly classified malignant images, 78 false-positive images, and 98 false-negative images. These values correspond to an overall accuracy of 97.12%, sensitivity of 96.44%, and specificity of 97.68%. Figure 7 presents the ROC curve, with an AUC-ROC of 0.9876 under the evaluated test setting.

### Grad-CAM visual explainability analysis

**Fig. 8:**
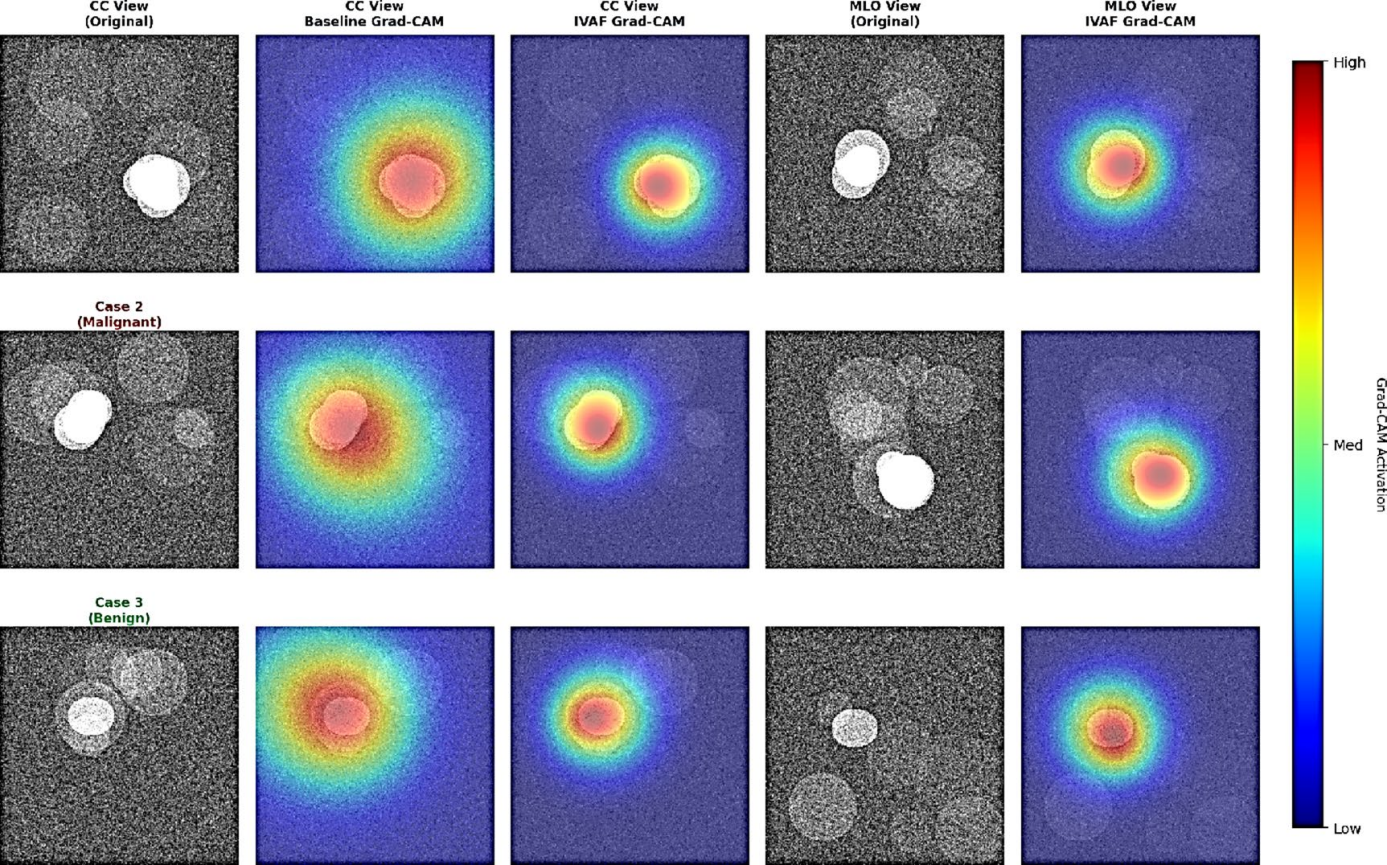
Grad-CAM visualizations for representative CBIS-DDSM test cases.

To provide qualitative insight into model attention, Gradient-weighted Class Activation Mapping (Grad-CAM) was applied to representative test cases using the fused feature tensor $\:{F}_{\mathrm{f}\mathrm{u}\mathrm{s}\mathrm{e}\mathrm{d}}$ generated by the IVAF module. Figure 8 compares baseline single-view ResNet50 activation maps with IVAF-based activation maps for paired CC and MLO projections.

In the representative examples shown, the baseline model produced broader activation regions, whereas the IVAF-based model produced more localized activation patterns around the lesion-centered region in both views. This observation is consistent with the intended role of IVAF as a cross-view fusion module. Nevertheless, Grad-CAM maps provide qualitative visual evidence of model attention and should not be interpreted as direct proof of diagnostic reliability, clinical significance, or radiologist-level interpretability. This cautious interpretation is consistent with recent reviews of explainable AI evaluation in mammography^34^. Expert radiologist assessment and external validation on independent datasets are required before stronger conclusions can be made regarding the clinical meaning of these activation patterns.

### Implications of patient-level partitioning

The emphasis on patient-level partitioning in this study is intended to improve transparency and reproducibility, not to question the validity of previous mammography classification studies. Because CBIS-DDSM contains multiple images, views, and annotations from the same patient, assigning all images from each patient to a single data partition is an important safeguard against patient-level overlap between training, validation, and testing.

In the present study, all images from the same patient were assigned to the same data split before augmentation. This procedure ensures that augmented training samples do not share patient-level anatomical information with validation or test images. Therefore, the reported results should be interpreted as performance under a clearly defined leakage-controlled CBIS-DDSM protocol rather than as a universal comparison against all previously published methods.

### Limitations and future research directions

Despite the encouraging performance of the developed system under the assessed CBIS-DDSM protocol, there are some aspects that should be taken into account as well. The first one is related to the fact that training and testing were performed only on CBIS-DDDM data, and therefore the results cannot be considered applicable for mammograms produced by other vendors, with different imaging protocol, other demographic structure of patients, and even for more modern techniques such as digital breast tomosynthesis and contrast-enhanced mammography. The second aspect is that Grad-CAM analysis shows qualitatively where the model focuses its attention but does not ensure the clinical interpretability of the system’s decisions. Expert assessment from radiologists is required to determine whether highlighted features have clinical significance. Finally, the test set consisted of 222 patients and 6,117 images.

External validation using different mammographic databases, e.g., INbreast or VinDr-Mammo, is needed to prove the applicability of the system in different acquisition conditions, at different institutions, and for different population of patients.

## Conclusion

This study presented a ResNet50-based breast cancer classification framework enhanced with an Inter-View Attention Fusion module for adaptive fusion of paired CC and MLO mammographic feature maps. The framework was evaluated on CBIS-DDSM using patient-level partitioning before augmentation, a two-stage transfer-learning strategy, component-wise ablation, repeated-seed stability analysis, and Grad-CAM-based qualitative visualization. Under the evaluated CBIS-DDSM setting, the model achieved an accuracy of 97.12%, sensitivity of 96.44%, specificity of 97.68%, and AUC-ROC of 0.9876 on the held-out test set. The ablation results indicate that selective fine-tuning, class-balanced loss weighting, post-split augmentation, and IVAF each contributed to final performance. However, because the current evaluation was limited to a single public dataset, these findings should be interpreted as promising dataset-specific evidence rather than definitive proof of broad clinical generalizability. External validation on independent mammography datasets and expert radiologist assessment remain necessary before considering broader clinical applicability.

## Data Availability

The paper has a dataset available in the Kaggle Repository, “https://www.kaggle.com/code/kerneler/starter-cbis-ddsm-breast-cancer-image-eef73d6f-4.

## Code availability

The source code used for preprocessing, training, evaluation, ablation analysis, and figure generation is available from the corresponding author upon reasonable request.
